# Bioinformatics Methods in Medical Genetics and Genomics

**DOI:** 10.3390/ijms21176224

**Published:** 2020-08-28

**Authors:** Yuriy L. Orlov, Ancha V. Baranova, Tatiana V. Tatarinova

**Affiliations:** 1 The Digital Health Institute, I.M. Sechenov First Moscow State Medical University of the Ministry of Health of the Russian Federation (Sechenov University), 119991 Moscow, Russia; 2 Life Sciences Department, Novosibirsk State University, 630090 Novosibirsk, Russia; 3 Institute of Cytology and Genetics SB RAS, 630090 Novosibirsk, Russia; 4 School of Systems Biology, George Mason University, Fairfax, VA 22030, USA; abaranov@gmu.edu; 5 Research Centre for Medical Genetics, 115522 Moscow, Russia; 6 La Verne University, La Verne, CA 91750, USA; ttatarinova@laverne.edu; 7 Department of Fundamental Biology and Biotechnology, Siberian Federal University, 660074 Krasnoyarsk, Russia

**Keywords:** genomics, bioinformatics, gene expression, medical genetics, human population genetics

## Abstract

Medical genomics relies on next-gen sequencing methods to decipher underlying molecular mechanisms of gene expression. This special issue collects materials originally presented at the “Centenary of Human Population Genetics” Conference-2019, in Moscow. Here we present some recent developments in computational methods tested on actual medical genetics problems dissected through genomics, transcriptomics and proteomics data analysis, gene networks, protein–protein interactions and biomedical literature mining. We have selected materials based on systems biology approaches, database mining. These methods and algorithms were discussed at the Digital Medical Forum-2019, organized by I.M. Sechenov First Moscow State Medical University presenting bioinformatics approaches for the drug targets discovery in cancer, its computational support, and digitalization of medical research, as well as at “Systems Biology and Bioinformatics”-2019 (SBB-2019) Young Scientists School in Novosibirsk, Russia. Selected recent advancements discussed at these events in the medical genomics and genetics areas are based on novel bioinformatics tools.

Computational models for molecular mechanisms gene expression regulation analysis are in high demand in biomedicine. Gene expression regulation could be controlled at transcriptional, post-transcriptional, translational, gene network and pathways levels. The series of post-conference special journal issues [1,2,3,4,5] started from Bioinformatics of Genome Regulation and Structure (BGRS) conferences and related schools on systems biology and bioinformatics (SBB) in Novosibirsk, Russia (http://conf.bionet.nsc.ru/sbb2019/en/). Human genomics applications were discussed at the “Centenary of Human Population Genetics” Conference 29–31 May 2019, and the Digital Medical Forum-2019 in Moscow (http://centenary-popgene.com/en). The papers joined this thematic issue on medical genomics beyond the conferences, suggesting an analysis of gene expression regulation and providing protein structure prediction tools. We start this paper collection from the systems biology models in oncology, complex diseases and drug analysis.

The paper by Marianna Zolotovskaia and co-authors [6] discovered heterogeneities of tumors and cross-analyzes them with repertoires of drugs, which are currently in use in clinical oncology along with their molecular targets. The tumors data were taken from The Cancer Genome Atlas database. For the first time, the authors showed that the repertoires of molecular targets of accepted drugs did not correlate with molecular heterogeneities of different cancer types. These findings provide a theoretical basis for reconsidering utilization of targeted therapeutics and intensifying drug repurposing efforts.

The work by Victor Tkachev et al. [7] showed the improvement of global machine learning methods in omics-based personalized oncology. Currently, Machine Learning (ML) methods are rarely used for an omics-based prescription of cancer drugs, due to a shortage of case histories with clinical outcomes supplemented by high-throughput molecular data. This causes overtraining and high vulnerability of most ML methods. The authors proposed a hybrid global–local approach to ML termed floating window projective separator (FloWPS) that avoids extrapolation in the feature space. Its core property is data trimming, i.e., sample-specific removal of irrelevant features. The computational experiments for 21 high-throughput gene expression datasets totally representing 1778 cancer patients with known responses on chemotherapy treatments showed the effectiveness of the method proposed. FloWPS essentially improved the quality of the treatment response classifiers for all global ML methods. Thus, FloWPS showed its robustness to overtraining.

The following papers discovered cases of genes the involvement of certain genes in phenotypes of complex diseases, such as HIV-1, autism and neurological diseases, and the discovery of relevant molecular targets.

The treatment of an HIV-1-positive patient requires that several drugs should be taken simultaneously. Olga Tarasova and colleagues [8] presented a computational approach for the prediction of the treatment and the effectiveness or failure of antiretroviral therapy. The resistance of the virus to an antiretroviral drug may lead to treatment failure. The approach focused on predicting the exposure of a particular viral variant to an antiretroviral drug or drug combination. The authors utilized nucleotide sequences of HIV-1 encoding protease and reverse transcriptase to perform such types of prediction. The Prediction of Activity Spectra for Substances (PASS) algorithm, based on the naive Bayesian classifier, was used to make a prediction. The probability of whether a sequence belonged or did not belong to the class associated with exposure of the viral sequence to the set of drugs can be associated with resistance to the set of drugs. High prediction accuracy for the prediction of treatment effectiveness was shown.

Autism spectrum disorder has a strong and complex genetic component with an estimate of more than 1000 genes implicated, cataloged in Simon’s Foundation Autism Research Initiative (SFARI) gene database. Notably, a significant part of both syndromic and idiopathic autism cases can be attributed to disorders caused by the mechanistic target of rapamycin (mTOR)-dependent translation deregulation. Ekaterina Trifonova and colleagues [9] presented a fundamental work of gene expression control in autism predisposition genes. The gene-set analyses allowed to find that 58% of the genes included in the SFARI gene database and 64% of the genes included in the first three categories of the database could be attributed to one of the four groups: fragile X mental retardation protein target genes; mTOR signaling network genes; mTOR-modulated genes; or vitamin D3 sensitive genes. The authors hypothesized that genetic and/or environment mTOR hyperactivation, including provocation by vitamin D deficiency, might be a common mechanism controlling the expressivity of most autism predisposition genes and even core symptoms of autism.

Tatiana V. Tatarinova and colleagues [10] analyzed therapy by MNRI^®^—Masgutova Neurosensorimotor Reflex Intervention. MNRI may facilitate neurodevelopment, build stress resiliency, neuroplasticity and optimal learning opportunity. The authors demonstrated that the MNRI approach is an intervention that reduces inflammation.

Several further works in this issue present novel bioinformatics methods and algorithms applicable for medical genomics and proteomics data.

The use of DNA microarrays for estimating miRNA expression profiles is limited by several factors including comparing expression values of different miRNAs. Stepan Nersisyan and co-authors [11] presented a post-processing algorithm for miRNA microarray data analysis. The algorithm performs the scoring of miRNAs in the results of microarray analysis based on expression values, time of discovery of miRNA and correlation level between the expressions of miRNA and corresponding pre-miRNA in considered samples. In this work, the authors show that the situation can be significantly improved if some additional information is taken into consideration in a comparison.

Valery Panyukov and co-authors [12] discussed the bioinformatics application to use k-mers in phylogenetic analysis and microbiome profiling. Alignment-free approaches based on the search for marker k-mers turned out to be capable of identifying not only species but also strains of microorganisms with known genomes. The authors evaluated the ability of genus-specific k-mers to distinguish eight phylogroups of *Escherichia coli* and assessed the presence of their unique 22-mers in clinical samples for patients with Crohn’s disease. The study proposes strain-specific “barcodes” for rapid phylotyping.

The affinity of different drug-like ligands to multiple protein targets is a subject of intense research. Nurbubu Moldogazieva and colleagues [13] presented modeling of protein binding sites for human alpha-fetoprotein. Alpha-fetoprotein (AFP) is a major embryo- and tumor-associated protein capable of binding and transporting a variety of hydrophobic ligands, including estrogens. The authors constructed a homology-based 3D model of human AFP with the purpose of the molecular docking of ERα ligands, three agonists (17β-estradiol and others) and three antagonists (tamoxifen, afimoxifene and endoxifen) into the obtained structure. Based on the ligand-docked scoring functions, three putative estrogen- and antiestrogen-binding sites with different ligand binding affinities were identified. 

Sergey Proshkin and co-authors [14] analyzed the human-specific isoform of RNA polymerase II. They experimentally estimated the interaction of RNA Polymerase II Subunit with the transcription factor ATF4. By a yeast two-hybrid screening of a human fetal brain cDNA library and subsequent co-purification assay in vitro, transcription factor ATF4 was identified as a prominent partner of the minor RNA polymerase II subunit hRPB11bα. In human RNA polymerase II that contains plural isoforms of the subunit hRPB11, the strength of the hRPB11–ATF4 interaction appeared to be isoform-specific, providing the first functional distinction between the previously discovered human forms of the Rpb11 subunit.

Dmitry Karasev et al. [15] showed the computational method for protein–ligand interaction predication. The affinity of different drug-like ligands to multiple protein targets reflects general chemical–biological interactions. The method proposed is based on the analysis of local sequence similarity within the set of analyzed proteins. The approach provides prediction accuracy comparable to or exceeding those of other methods, as it was demonstrated on the popular Gold Standard test sets. Thus, the method can be applied to the broad area of protein–ligand interactions.

To modify chromatin, long noncoding RNA (lncRNA) often interacts with DNA in a sequence-specific manner forming RNA: DNA triple helices. Elena Matveishina and co-authors [16] compared bioinformatics tools for RNA:DNA triple helix prediction. Computational tools for a triple helix search do not always provide genome-wide predictions of sufficient quality. The authors used four human lncRNAs (MEG3, DACOR1, TERC and HOTAIR) and their experimentally determined binding regions for evaluating triplex parameters that provide the highest prediction accuracy. The science team combined triplex prediction with the lncRNA secondary structure and demonstrated that considering only single-stranded fragments of lncRNA can further improve DNA-RNA triplexes prediction.

The following articles initially discussed at the “Centenary of Human Population Genetics” Conference (http://centenary-popgene.com/en) highlight the problems of human population genetics and their solutions achieved by genomics data analysis.

Rena Zinchenko and colleagues [17] studied the allelic heterogeneity of hereditary diseases in human populations. The study presented the results of a genetic epidemiological study of hereditary diseases in the population of the Karachay-Cherkess Republic. Frequent diseases were determined; the presence of marked genetic heterogeneity was identified during the confirmatory DNA diagnosis. Correlation analysis showed that genetic drift is probably one of the leading factors determining the differentiation of the populations studied by hereditary disease load.

Viola Grugni and co-authors [18] analyzed human populations in Sardinia. Many anthropological, linguistic, genetic and genomic analyses have been carried out to evaluate the potential impact that evolutionary forces had in shaping the present-day Sardinian gene pool, the main outlier in the genetic landscape of Europe. The authors analyzed the male-specific region of the Y chromosome in three population samples obtained by reallocating a large number of Sardinian subjects to the place of origin of their monophyletic surnames, which are paternally transmitted through generations in most of the populations, much like the Y chromosome. The results show that the analysis of the Y chromosome gene pool coupled with a sampling method based on the origin of the family name is an efficient approach to unraveling past heterogeneity, often hidden by recent movements, in the gene pool of modern populations. 

The work by Mikhail Ponomarenko et al. [19] considered nucleotide polymorphisms in the human genome regulating gene expression. Susceptibility to atherogenesis-associated diseases is caused by single-nucleotide polymorphisms (SNPs). Atherosclerosis-related myocardial infarction and stroke remain the main causes of death in humans. Using the previously developed public web-service SNP_TATA_Comparator, the authors estimated the statistical significance of the SNP-caused alterations in TATA-binding protein for their binding affinity for proximal promoter regions of the human genes clinically associated with diseases either syntonic or dystonic with atherogenesis. The results uncovered SNPs near clinical SNP markers as the basis of neutral drift accelerating atherogenesis and SNPs of genes encoding proteins related to mitochondrial genome integrity and microRNA genes associated with instability of the atherosclerotic plaque as a basis of directional natural selection slowing atherogenesis. Note the related bioinformatics tools papers [20,21] published in the *Frontiers in Genetics* special issue “Bioinformatics of Genome Regulation and Systems Biology” [22], and *BMC Genomics* issue [23]. The research topic on gene expression regulation in *Frontiers in Genetics* is continued in 2020.

The guest editors are happy to announce the next post-conference journal issue at MDPI IJMS for the BGRS\SB-2020 conference (https://bgrssb.icgbio.ru/2020/) in Novosibirsk, Russia (https://www.mdpi.com/journal/ijms/special_issues/Bioinformatics_Genomics) as well as to extend the current medical genomics papers collection by the new “Medical Genetics, Genomics and Bioinformatics-2020” issue (https://www.mdpi.com/journal/ijms/special_issues/Medical_Genetics_Bioinformatics_2). A new Special Issue will collect papers on medical genomics, human population genetics and computational biology applications in biomedicine, providing a continuation of this MDPI IJMS Special Issue. Based on the readers’ interest in medical genetics and genomics, we are continuing our publication in this science area based on novel technological approaches, gene networks and metabolic pathways analysis.
